# Environmental and neurodevelopmental contributors to youth mental illness

**DOI:** 10.1038/s41386-024-01926-y

**Published:** 2024-07-19

**Authors:** Sarah Whittle, Lu Zhang, Divyangana Rakesh

**Affiliations:** 1 Centre for Youth Mental Health, The University of Melbourne, Parkville, VIC Australia; 2 Orygen, Parkville, VIC Australia; 3 Neuroimaging Department, Institute of Psychology, Psychiatry & Neuroscience, King’s College London, London, UK

**Keywords:** Stress and resilience, Development of the nervous system

## Abstract

While a myriad of factors likely contribute to the development of mental illness in young people, the social environment (including early adverse experiences) in concert with neurodevelopmental alterations is undeniably important. A number of influential theories make predictions about how and why neurodevelopmental alterations may mediate or moderate the effects of the social environment on the emergence of mental illness. Here, we discuss current evidence supporting each of these theories. Although this area of research is rapidly growing, the body of evidence is still relatively limited. However, there exist some consistent findings, including increased striatal reactivity during positive affective processing and larger hippocampal volumes being associated with increased vulnerability or susceptibility to the effects of social environments on internalizing symptoms. Limited longitudinal work has investigated neurodevelopmental mechanisms linking the social environment with mental health. Drawing from human research and insights from animal studies, we propose an integrated mediation-moderation model and outline future research directions to advance the field.

## Introduction

The social environment, particularly experiences of adversity and stress, has profound effects on cognitive, social, and emotional functioning in young people. Childhood adversity is one of the strongest predictors of mental illness onset [1], particularly during the first two decades of life when many mental illnesses first emerge [2]. During this time, the brain is rapidly developing, and alterations in brain development are also thought to contribute to the onset of mental illness [3, 4]. With the ultimate goal of developing better prevention and intervention strategies to reduce the immense burden of child and adolescent mental illness, which has increased substantially in recent decades [5], research and theory have increasingly sought to better understand the role of both neurobiological and environmental factors in mental illness risk. To this end, the aim of the present review is to discuss theories that explore how the dynamics between the social environment and neurobiology intersect to shape the emergence of mental illness in young people. Drawing upon available evidence, with a specific emphasis on human neuroimaging studies, we aim to provide insights into the validity of these theories. We conclude with recommendations for a more integrated theoretical model and suggest avenues for future research in this area.

A key insight from developmental neuroscience, relevant to understanding the integrative role of the social environment and the brain in mental health and illness, is brain plasticity. Brain plasticity, which is substantially higher during childhood and adolescence relative to adulthood, refers to the ability of the brain to reorganize neural pathways based on input from the environment [6]. The overproduction and elimination of immature synapses during childhood and adolescence, driven by both intrinsic programming and environmental influences, are thought to be key mechanisms of plasticity [6, 7]. However, other mechanisms include the overproduction of neurons in early development, apoptosis or programmed cell death of excessive neurons, and at the molecular level, central nervous system receptor alterations [6, 7].

Of particular relevance to the development of mental illness in young people is the notion that social environmental exposures during periods of increased brain plasticity may alter neurodevelopment. Altered neurodevelopment may, in turn, increase or decrease susceptibility to the development of mental health problems. Such environmental input may have these effects via experience-expectant or experience-dependent processes [8, 9]. Experience-expectant processes refer to changes resulting from the presence or absence of environmental stimuli that the neural system has evolved to expect early in life (e.g., the presence of a sensitive and responsive caregiver vs. emotional neglect, provision of food and shelter vs. physical neglect, exposure to sensory and linguistic experiences vs. deprivation of such experiences) [10]. During such periods, expected inputs are thought to have an increased impact on brain organization, triggering periods of increased plasticity (i.e., sensitive periods). Conversely, experience-dependent plasticity refers to the brain’s ability to reorganize and adapt in response to individual experiences and environmental stimuli. Experience-dependent plasticity is thought to occur throughout the lifespan but diminishes as the brain matures. Adverse experiences that contribute to experience-dependent plasticity might include the presence of traumatic or stressful stimuli/events that threaten or are perceived to threaten (from an evolutionary standpoint) survival (e.g., physical or sexual abuse, exposure to crime, domestic violence, natural disaster, social exclusion). Positive social experiences that may also impact neurodevelopment via experience-dependent mechanisms include supportive family and peer relationships [11–13], exposure to stimulating educational settings [14, 15], etc.

Building on the notion of plasticity, a number of theories and models have emerged that consider the role of neurodevelopmental processes in the link between the social environment and the emergence of mental illness during childhood and adolescence. These theories serve to provide testable hypotheses, direct research efforts and move the field forward to advance knowledge, with the ultimate goal of clinical and policy translation. These theories differ in their developmental versus evolutionary focus, whether they take a deficit versus strengths-based approach, and whether they focus on adaptive versus maladaptive processes. They also differ in whether they primarily focus on neurobiology or neurodevelopment as a mechanism (i.e., mediator) versus a moderator of environmental-mental health associations (see Box 1). We next discuss these theories and their current empirical evidence, grouped by whether they take a mediation versus moderation approach.

Box 1 Statistical mediation versus moderation Two statistical concepts, namely mediation and moderation, are commonly used to elucidate the complexities of relationships among three or more variables. Statistical mediation tests whether and to what extent an independent variable (IV) influences a dependent variable (DV) via one or more intervening variables. It thereby tests mechanistic questions, explicating the underlying causal pathway between the IV and DV, or the “how” and “why” an IV influences the DV. An intervening variable or variables may partially or fully mediate (or explain) the association between the IV and DV. Further, contemporary theory suggests that a statistically significant association (or a total effect) does not necessarily need to exist between the IV and DV for statistical mediation to be possible [103, 104]. This is because there can be many different paths of influence from the IV to the DV, and these paths could operate in opposite directions, canceling each other out and producing an overall non-significant total effect between the IV and DV. To provide an example of a mediation effect, if time spent studying significantly mediates the association between intelligence quotient (IV) and exam performance (DV), then this may indicate that part of the effect of intelligence quotient on exam performance operates through the amount of time spent studying. Students with higher intelligence quotients may be more likely to study longer, leading to better exam performance.

In contrast, statistical moderation refers to the conditional nature of the relationship between an IV and DV, contingent upon the levels of a third moderating variable (or moderating variables). By examining the interactive effects of IV and moderator/s in predicting the DV, moderation analysis unpacks the nuanced conditions under which the relationship between IV and DV intensifies, weakens, or remains unchanged depending on the moderating variable/s. In the case of significant moderation, the association between an IV and DV may be stronger, or even only present, at a certain level of a (categorical or continuous) moderating variable. Or it could be strong but a different sign/direction (positive versus negative) for different (e.g., high versus low) levels of a moderator. Using the same variables from the mediation example, if time spent studying significantly *moderates* the association between intelligence quotient (IV) and exam performance (DV), the effect of intelligence quotient on exam performance varies depending on the amount of time spent studying. For students who study less, a higher intelligence quotient may lead to superior exam performance. For those who study more, intelligence quotient may not be associated with exam performance.

## Models of neurodevelopmental mechanisms linking the social environment with psychopathology (i.e., mediation models)

Theories aiming to explain neurodevelopmental mechanisms linking adverse social environmental experiences with mental health propose a causal chain whereby adverse experiences directly impact the developing brain, which in turn increases the risk of developing psychopathology. One such theory is the *Stress Acceleration Hypothesis* [SAH [16], which builds on evolutionary theories [including Life History Theory [17] that posit faster biological development to be an evolutionary adaptive strategy that allows for earlier reproduction in contexts of adversity. The SAH suggests that adverse experiences (particularly relating to the caregiving environment) accelerate neurodevelopmental processes to reach “adult-like” functioning earlier. Specifically, the hypothesis refers to the accelerated development of brain regions and connections considered important for emotion processing, emotion regulation, and memory (specifically, amygdala-prefrontal circuitry); because in situations defined by missing or inconsistent parental care, it may be adaptive to transition from a state of parent-regulation to self-regulation sooner. The SAH also argues that the precocious development of this circuitry, while initially adaptive, could contribute to impairment in emotion regulation abilities, thus having negative consequences for later mental health.

Although the SAH originally posited for this acceleration to occur in the context of caregiver-related adversity, this model has been extended and applied more broadly to early adverse experiences characterized by chronic stress [18]. Tooley et al. argue that chronic stress (which may occur, for example, in the context of socioeconomic disadvantage) may lead to accelerated brain development via the process described by the SAH, but other mechanisms may include repeated use of stress-regulation circuitry, and/or via increasing glucocorticoid levels and activation of inflammatory processes [18]. Specific neural circuits are not specified, however, and predictions are made about brain development in general. Rakesh et al. [19], similarly speculating about both adversity and brain development more generally, have suggested that adversity (specifically low socioeconomic status) is likely to be associated with a distinct developmental pattern involving slower brain development across childhood and adolescence. They make this prediction based on longitudinal evidence of socioeconomic disadvantage being associated with a slower rate-of-change and persistently lower cortical thickness, area, and volume throughout childhood and adolescence [19], and suggest that these brain changes mediate links with poor mental health (and other) outcomes. Specific mechanisms are suggested to include impaired synaptic pruning and slower neural circuit refinement. Another theory with similarities to SAH, the *latent vulnerability theory* [20], suggests that early maltreatment creates an underlying latent vulnerability, increasing future mental health risk. This latent vulnerability is suggested to involve alterations to one or more of several neural systems, including those that modulate threat processing, reward-processing, executive control, and emotion regulation. Notably, this theory does not specify that these alterations necessarily reflect accelerated (or delayed) neural development.

Another extension of the SAH was proposed by Colich et al. [21], who suggested that stress-associated acceleration may depend on the type of adversity experienced, in line with the *Dimensional Model of Adversity and Psychopathology* (DMAP) [22–24]. The DMAP proposes that adversity can be classified into two main types: threat and deprivation. Experiences of threat and harm (such as physical, sexual, and emotional abuse) can be classified as “threat”, whereas the lack of expected caring and nurturing input (e.g., lack of physical and emotional support, and cognitive/social stimulation) can be classified as experiences of deprivation. The theory posits that while they often co-occur, threat and deprivation have at least partially distinct neurodevelopmental correlates. Colich et al. [21], similarly to Tooley et al. and Rakesh et al., went beyond the idea of acceleration of the amygdala-prefrontal circuit, and discussed the idea of biological aging more globally. They proposed the idea that experiences characterized by threat, but not deprivation, would be associated with accelerated biological aging, including accelerated gray matter and amygdala-prefrontal connectivity development. A recent *integrated model of dimensions of environmental experience* [25] builds on these ideas further, suggesting that deprivation is likely to have complex effects, particularly relating to whether it is predictable vs. unpredictable, or within proximal vs. distal environments. For example, it is suggested that when experiences of deprivation are unpredictable, the neurobiology supporting some aspects of executive functioning (e.g., inhibitory control, working memory capacity) will show a delayed development, while alterations to other aspects may be mitigated in the case where these functions are important for survival (e.g., cognitive flexibility allows for flexibly switching between mental sets).

The recently developed *change of pace* theory [26] provides a similar account to the aforementioned models, insofar as it proposes different neurodevelopmental trajectories depending on specific forms of adversity experienced in childhood. The *change of pace* hypothesis contends that the type of adversity encountered determines whether biological maturation is expedited or retarded by focusing on the parent-child dyad, and suggests that changes in the rate of development occur primarily to eliminate gaps in parental caregiving. They purport that delaying maturation specifically lowers children’s total physiological requirements when there are unmet physiological needs, like in situations of deprivation or neglect (e.g., in case of inadequate nutrition or parental neglect). In the event of threat or abuse however, if there are unmet safety needs (e.g., shelter from threat or protection), accelerated development would boost children’s general ability for autonomous safety requirements provision, with this acceleration resulting in a later (post-childhood) stunting of developmental autonomy. While *change of pace* theory does not provide a comprehensive account of neural circuits implicated, the function and neural regulation of the HPA-axis is discussed as likely critical.

## Empirical evidence for mediation models from longitudinal studies

While others have reviewed evidence for the aforementioned models, they have included both cross-sectional and longitudinal studies [18, 21] or have not focused on studies investigating mental health outcomes using statistical tests for mediation (e.g., [19]). Given that most of these models make predictions about developmental trajectories, cross-sectional data is arguably inappropriate for testing them [19]. This is not to say that cross-sectional examinations of brain structure, function or connectivity cannot provide useful information about the impact of environmental exposures on development. Rather, longitudinal research is needed to truly test deviations from typical developmental trajectories as proposed by the aforementioned models. Further, studies investigating associations between adverse social environments and brain development, without investigating links with mental health outcomes, are useful for testing these models (e.g., as reviewed by [18, 19, 21]). Here, however, we focus only on studies that investigated mental health outcomes using statistical tests of mediation, so that interpretation of the adaptive versus maladaptive nature of adverse environmental impacts on brain development was possible. As such, we exclusively review studies investigating *longitudinal neurobiological mediators* of the associations between adverse social environmental variables and mental health outcomes. Finally, we did not restrict our review of studies based on the specific type of adverse social environment assessed.

Some support for the SAH, Tooley et al., and the *latent vulnerability theory* has come from studies investigating longitudinal resting-state connectivity changes in adolescents. Links identified with mental health point to both adaptive and maladaptive neurobiological changes following adversity. Rakesh et al. [27], for example, found both childhood abuse and neglect to be associated with increases in within-salience network (SN) resting-state connectivity between ages 16 to 19, and this pattern of connectivity mediated associations with lower problematic substance use *and* higher depressive symptoms. This pattern of connectivity was suggested to reflect more advanced, or accelerated, development of circuitry responsible for the evaluation of internal and external states, thus consistent with the SAH/Tooley et al. The involvement of a network responsible for the detection of salient affective stimuli is consistent with *latent vulnerability* theory. The links identified with different mental health outcomes were intriguing. Given the role of the SN in reward processing [28], Rakesh et al. suggested that maltreatment-associated increases in SN connectivity may be associated with reduced reward sensitivity, which may decrease the likelihood of adolescents engaging in risky but rewarding behaviors such as substance use, and at the same time increase the risk for depression, which is associated with dampened reward function [29].

There have been similar reports of adversity-related alterations in resting-state connectivity development being associated with more positive mental health outcomes. For example, Brieant et al. [30] showed that lifetime negative life experiences were linked to reductions in cortico-limbic resting-state functional connectivity over time during early adolescence (from age 9 to 13), which, in turn, was associated with lower internalizing symptoms. Based on normative patterns of connectivity development, the authors suggest that this finding reflects accelerated maturation of neural circuitry responsible for emotion processing and regulation (consistent with SAH/Tooley et al./*latent vulnerability* theory), which is adaptive and beneficial for wellbeing. Further discussion on the possibility of these neurobiological alterations reflecting adaptive brain phenotypes is provided in subsequent sections.

In alignment with the *integrated model of dimensions of environmental experience* and *change of pace* theory, Rakesh et al. [31] found that increases in connectivity between cortical networks (particularly the default mode, frontoparietal, dorsal attention and salience networks) from age 16 to 19 mediated the relationship between childhood maltreatment and later depressive symptoms, with findings potentially driven by experiences of neglect. Given that between-network connectivity normatively decreases across adolescence, and increased connectivity between functionally distinct networks has been linked to poor cognitive performance [32], the authors suggested that findings may reflect a mechanism by which delayed functional connectivity development underlies cognitive impairments in adolescents with depression and a history of deprivation. Similarly, Hanson et al. [33] found that emotional neglect was associated with blunted development of reward-related ventral striatal activity between early and late adolescence (approximately 11–17 years of age). Additionally, blunted reward-related ventral striatal activity mediated the association between neglect and greater depressive symptoms approximately 1 year later. This finding may be interpreted to reflect delayed development of reward-related neural circuitry, which may be particularly relevant to symptoms of anhedonia associated with depression.

Regarding brain structural development, two studies have implicated hippocampal development in patterns consistent with different models. Miller et al. [34] found that greater perinatal adversity (e.g., low birth weight, low family income, maternal mental health problems) was associated with increased growth in the right hippocampal body across childhood (from age 4.5 to 7.5). Further, adversity was indirectly associated with late childhood (age 8.5) depressive symptoms via increased hippocampal body growth. Conversely, Barch et al. [35] found that early childhood poverty was related to reduced testosterone increases. This was, in turn, linked to smaller initial volume and reduced volumetric growth of the hippocampus during the transition from school age to adolescence (i.e., age 7 to 12). These neurobiological changes mediated associations with elevated emotion dysregulation and depressive symptoms during late adolescence (age ~19). As such, these two studies identify opposite findings of increased and reduced hippocampal development potentially reflecting maladaptive patterns of development contributing to increased vulnerability to the development of depression. In the former case, findings may reflect accelerated development following adversity, consistent with SAH/Tooley et al., whereas in the latter case, findings may reflect a delay in development, consistent with Rakesh et al.’s model, *integrated model of dimensions of environmental experience*, and *change of pace* theories (to the extent that poverty may reflect deprivation experiences).

While these studies provide some support for the aforementioned mediation models, there are a number of examples of relevant studies with null findings [36–41], clearly highlighting the need for further research in this area. Future work should also address limitations of the current evidence base. In particular, in many of the reviewed studies, conclusions about accelerated or delayed neurobiological development are limited, given (1) studies do not examine the full span of neurodevelopment (from birth to adulthood), (2) normative patterns of neurodevelopment (from which to compare) are not necessarily known (particularly for measures of brain function or functional connectivity), and (3) non-linear growth is rarely investigated [despite the normative non-linear brain developmental patterns for a number of neural metrics [42]. It is important to note, however, that a few studies exist that address some of these issues using methods other than mediation to test links with mental health [43].

## Models of neurodevelopmental factors that modify associations between social environmental factors and mental health (i.e., moderation models)

Person-by-environment interaction frameworks are commonly used to understand individual risk or susceptibility to mental health problems. Such conceptual models have a common tenet that people are variably influenced by environmental factors, and one source of this variability is likely person-centered factors. These include biologically-based person-centered factors, including aspects of brain structure and function. Within the literature, there are two broad conceptualizations of person-by-environment or biology-by-environment interactions. *Developmental* theories purport that there is an optimal trajectory of human development that adversity can derail. *Evolutionary-developmental* theories argue against the existence of a single optimal trajectory of development. Instead, they posit that because humans have evolved to develop in various environments, both adverse and supportive, there may be multiple adaptive developmental trajectories. In this sense, optimal development is framed as context-dependent. Notably, these theories are not particularly specific regarding the types of relevant social environmental factors; rather, there is a general focus on early social environments that are either negative/adverse/marked by chronic stress or positive/supportive/low stress. While we do not cover all relevant models here, we focus on key models where measures of neurobiology have been utilized to examine interactions between biology and environment in relation to psychopathology in children and adolescents.

The broad class of developmental theories includes the *dual-risk model* [44], *diathesis-stress* theory [45], and *cumulative stress* theory [46]. These models generally argue that some individuals possess inherent vulnerabilities to poor developmental outcomes including psychopathology, which can be triggered by environmental stressors. *Cumulative stress* theory suggests that if there is an accumulation of stressors across the lifespan that exceeds a certain threshold, at-risk individuals are more likely to develop psychopathology [46]. Most formulations of these models suggest that the inherent vulnerability (or diathesis) factors are biological [47–49].

Evolutionary-developmental models, including the *Differential Susceptibility* model [50] and the *biological sensitivity to context* model [51], argue that individuals vary in their susceptibility to environmental influence, for better and worse. That is, highly susceptible individuals benefit more from supportive environments and suffer more in adverse circumstances compared to those with low susceptibility. *Differential susceptibility* developed from evolutionary theory [50], and *biological sensitivity to context* was formed in response to unexpected findings revealing that children with high immune reactivity exhibited the highest rates of illness in high-stress family environments and lowest illness occurrence in low-stress environments [51, 52]. Together, these frameworks, hereafter referred to as “differential susceptibility” due to their substantial overlap [53, 54], argue that susceptibility is an evolutionary-adaptive characteristic that is instantiated biologically early in life through genetics, intermediate phenotypes (e.g., the brain), and behavioral phenotypes. Differential susceptibility theory does not discount that dual-risk/diathesis-stress processes exist; rather, it argues that the same biological factor could lead to different outcomes in different contexts. If supportive environments and positive outcomes are not investigated, then differential susceptibility effects may be overlooked and mislabeled as vulnerability effects [55].

Extension to these theories include the *match*/*mismatch hypothesis*, which stems from evolution theory, and suggests that individuals with high “programming sensitivity” are more likely to be at mental health risk if a mismatch occurs between the early programming environment and the later adult environment. The benefits and costs of high programming sensitivity (which might take the form of higher physiological reactivity, for example) are context-dependent. Individuals with high programming sensitivity adapt to early environments in preparation for similar future experiences. If a context is encountered in the future that does not match the early environment, then there may be poor mental health outcomes for the individual. These individuals, however, are suggested to benefit from a match of early life and adult environment, even if both are aversive [56]. A similar idea was captured by the *3*-*hit hypothesis* (where the first “hit” represents a specific genetic background) [57].

## Empirical evidence for moderation models

The neural mechanisms underlying vulnerability or sensitivity to the social environment have been increasingly investigated by researchers interested in testing biology-by-environment interactions. Below, we review studies that have sought to test *dual-risk*/*diathesis-stress* and *differential susceptibility* theories, as currently there is limited human empirical work that has sought to test the nuances of some of the other theories. Notably, we only review studies that test the interaction between a social environmental variable and brain structure of function in association with mental health. Cross-sectional or longitudinal studies are reviewed given that these theories make no predictions about brain development per se. Again, we do not constrain our review of studies based on the negative or positive social environmental variables investigated.

A number of studies have investigated neural function during exposure to positive or rewarding stimuli in adolescents (e.g., [58–60]). Most results have supported dual-risk/diathesis-stress effects, with greater striatal activation to such stimuli being associated with higher internalizing [59, 61] or externalizing [60] symptoms in the context of adverse environments (e.g., high family conflict, [60, 61]; peer victimization [59]). These results may be interpreted as indicating that youth who are highly (neurally) sensitive to reward may be highly tuned to social interactions with friends and family. As a result, they may be more likely to be negatively affected by adverse family and peer environments, and more vulnerable to developing mental health problems as a result.

Others have found support for neural reactivity to positive stimuli/reward as reflecting differential susceptibility. Liu et al. [58], for example, formally (statistically) tested for differential susceptibility effects, and found that heightened amygdala reactivity to positive stimuli correlated with negative outcomes, such as increased internalizing symptoms and reduced prosocial behavior, in environments marked by negative family dynamics, such as low maternal warmth or imbalanced family functioning. Conversely, increased amygdala reactivity was associated with better outcomes, including lower symptom levels and greater prosocial behavior, in positive family settings characterized by high maternal warmth and balanced family functioning. Therefore, amygdala reactivity to positive stimuli may indicate differential susceptibility (rather than vulnerability alone) to the family environment.

Fewer studies have investigated neural activation associated with negative emotion processing (e.g., [62–64]) or other aspects of neural function (e.g., cognitive functioning [65]). These studies, conducted in late-childhood and adolescent samples, largely report findings consistent with dual-risk/diathesis-stress, with alterations in the function of regions and networks including the anterior cingulate cortex [63], inferior parietal sulcus [62], posterior cingulate, temporoparietal junction and amygdala [64], and executive network [65] being associated with increased externalizing problems when exposed to negative social environments (including low parental support [62], family disconnection [63], neighborhood crime [64] and neighborhood threat [65]).

A few studies have found patterns of resting-state connectivity to be associated with vulnerability to internalizing symptoms in adolescents, consistent with dual-risk/diathesis-stress. For example, lower coupling between the amygdala and vmPFC [66] and higher initial but steeper decreases in average whole-brain connectivity from age 9 to 19 years [67], have been associated with higher internalizing symptoms in the context of more averse environments (stressful life events [66], unpredictable negative life events [67]). In another study, lower connectivity in the central executive network and higher anti-correlation between the salience and default mode networks were associated with differential susceptibility in adolescents, whereby for adolescents with these patterns of connectivity, high maternal hostility was associated with higher anxiety symptoms, whereas low maternal hostility was associated with lower anxiety symptoms [68].

A number of studies have investigated cortical and subcortical structure as possible diathesis or susceptibility markers [69–77]. Most studies have focused on the adolescent period, while some have investigated infants [71, 73]. The hippocampus has been particularly implicated in these studies, with several studies of adolescents suggesting that larger hippocampal volume may confer vulnerability or perhaps susceptibility to environmental exposures, being associated with greater depressive symptoms in the context of more negative environments (high family conflict, family disconnection, high community crime, high levels of aggressive parenting behavior) and fewer depressive symptoms in the context of more positive environments (low family conflict, family connection, low community crime, low levels of aggressive parenting behavior) [70, 74, 75]. As suggested by Schriber et al. [74], given the role of the hippocampus in complex contextual memory processing [78], and findings of larger hippocampal volumes being associated with superior episodic learning and memory ability [79], findings may suggest that adolescents with larger hippocampal volumes process their social contexts more deeply, rendering them more susceptible to the influences of both positive and negative experiences. Notably, larger hippocampal volumes were found in one study of infants to reflect a susceptibility marker to maternal sensitivity in relation to attachment outcomes [73], indicating that larger hippocampal volumes may be a susceptibility marker across different developmental periods. Also notable is that there is some evidence for sex differences in this effect, with one study reporting a susceptibility effect for females but not males [75]. The interpretation of this sex difference is unclear but could reflect sex differences in neurodevelopmental trajectories leading to sex differences in specific periods of neuroplasticity.

## Summary of support for models

Although not a systematic review of the literature, the studies discussed do offer some degree of support for many of the discussed models. Two relatively consistent findings emerged. Increased striatal reactivity to positive/rewarding stimuli appeared to reflect a vulnerability or diathesis, associated with high internalizing problems in the context of a negative social environment. Larger hippocampal volumes appeared to reflect a susceptibility marker, related to higher internalizing problems in the context of a negative social environment and fewer problems in the context of a positive social environment. There are currently too few longitudinal studies, however, to make strong conclusions about consistent effects of mediation models. Below we discuss how the reviewed theories and models may be integrated to guide future research and move the field forward.

## Integration of models

The aforementioned theories and models are not mutually exclusive. It is possible that they all accurately describe aspects of the complex role of neurobiology in linking the social environment with mental health. It is also possible, if not very likely, that the environment shapes neurodevelopment, *and* patterns of neurodevelopment determine ongoing vulnerability or susceptibility to environmental exposures. It is important to point out that the descriptions of the theories provided above are relatively brief, and some of the theories provide far more complex accounts that provide a basis for integration across mediation and moderation models. For example, *biological sensitivity to context* theory suggests that increased sensitivity is driven by early environmental exposures, such that those growing up in more supportive *or* more adverse early environments are hypothesized to become more sensitive to future environments. In the former case, it would be adaptive to be maximally influenced by the developmental environment, and in the latter case, it would be adaptive to be vigilant to threats in order to increase chances of survival and eventual reproduction [53]. *Latent vulnerability theory* suggests that one of the ways that latent vulnerabilities, induced by early experiences of maltreatment, may increase risk is by altering responses to future stressors [20].

Few human neuroimaging studies have attempted to test such complex models involving neurobiology as both a mechanism/mediator and moderator of associations between the social environment and mental health outcomes, although we note one recent study that investigated prenatal adversity [80]. In this study, prenatal adversity (e.g., prenatal maternal mental health problems and poverty) was associated with a steeper decrease in structure-function connectivity coupling (SC-FC) from early- to mid-childhood, potentially suggestive of accelerated neurodevelopment. SC-FC during early childhood moderated the association between prenatal adversity and both internalizing and externalizing problems during mid-childhood. Specifically, high early childhood SC-FC appeared to act as a diathesis, such that only children with high SC-FC experienced poor mental health in the context of high prenatal adversity.

**Fig. 1:**
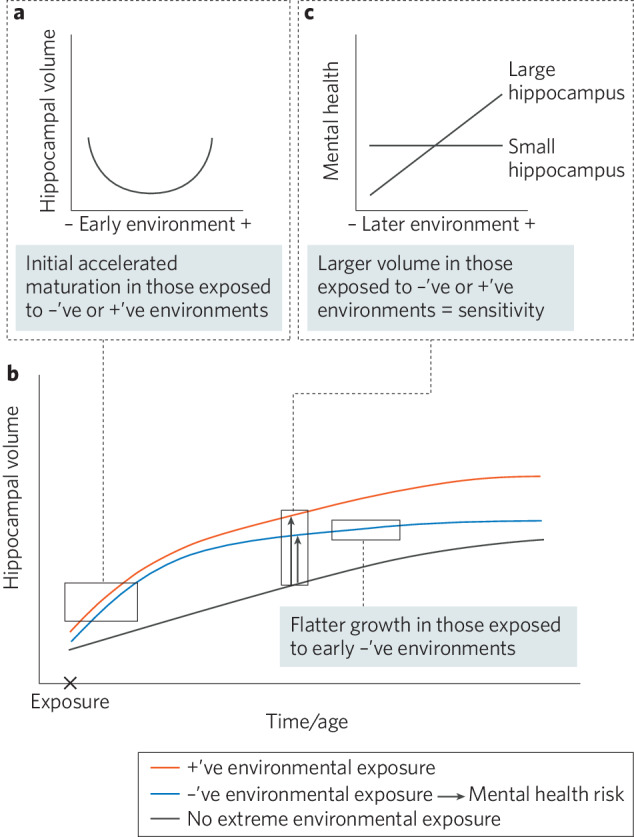
Integrated mediation-moderation model of the role of neurodevelopment in linking the social environment with mental health. +’ve = positive environmental exposure, −’ve = negative or adverse environmental exposure. **a** During early life, both positive and adverse environments lead to accelerated hippocampal maturation, resulting in a nonlinear association between the social environment and hippocampal volume. **b** Adverse early environments lead to early maturation, followed by a stunting of hippocampal growth during childhood and adolescence, increasing risk for mental health problems. **c** Larger hippocampal volumes following early accelerated hippocampal maturation engender increased sensitivity to later positive and adverse environments.

Here, we build on the reviewed theories and models, and together with insights from the reviewed studies, we propose an integrated mediation-moderation model of the role of neurodevelopment in linking the social environment with mental health (Fig. 1). We focus on adversity associated with the experiences of stress, but do not make predictions about specific dimensions of adversity. We make specific predictions in relation to hippocampal volume. However, we also discuss how the model may extend more broadly to neural circuitry supporting the detection and processing of salient affective stimuli.

We suggest that both positive and negative early environments may drive neurobiological changes that increase sensitivity to future environments, consistent with predictions from the *biological sensitivity to context* theory [53]. This may involve accelerated maturation of the hippocampus, to support increased contextual learning and memory processing [81]. Accelerated maturation of the hippocampus following early adversity is consistent with Miller et al.’s [34] finding (reviewed above) of perinatal adversity being associated with increased growth in hippocampal volume across early childhood. Accelerated maturation of neural systems supporting emotion and memory processing following adverse environments is also consistent with the SAH. We suggest that exposure to particularly *positive* environments may similarly lead to accelerated hippocampal maturation. Such a notion is consistent with animal research showing that both stressful *and* enriching early environments result in neurobiological markers of accelerated maturation in the hippocampus [82, 83]. This early accelerated maturation resulting in relatively larger hippocampal volumes, might persist throughout development, rendering individuals continuously sensitive to positive and negative environments—a concept consistent with *differential susceptibility* or *biological sensitivity to context*. This proposition is also consistent with the reviewed findings: larger hippocampal volumes in both infancy and adolescence were associated with increased susceptibility to both negative and positive environments; in the context of more negative environments, those with larger hippocampal volumes experienced internalizing symptoms, but in the context of positive environments, these individuals experienced fewer symptoms than their less susceptible peers [70, 73–75].

Following initial accelerated maturation in response to early *adverse* environments, we propose that hippocampal growth may slow after a period of time, due to the effects of the early adversity and associated stress on growth programs. In those exposed to early positive environments, growth subsequent to initial acceleration is not affected. The notion of early adversity being linked with an initial accelerated maturation of the hippocampus, combined with stunting of its growth, is consistent with the *change of pace* theory predictions about development following threat to safety needs. It is also consistent with animal work. Specifically, animal work has shown that early adversity or stress leads to markers of accelerated hippocampal maturation (precocious arrival of Parvalbumin-positive cells, earlier switch in NMDA receptor subunit expression, earlier rise in myelin basic protein expression), combined with markers of reduced hippocampal growth (earlier developmental decline in expression of markers of cell proliferation and differentiation) [82]. Accelerated maturation may be adaptive and have short-term advantages, but at the expense of truncated growth, leading to longer-term poor outcomes and risk for mental health problems. As reviewed above, Barch et al. [35] found early adversity (poverty) to be associated with reduced hippocampal growth from late childhood through adolescence, and this reduced growth was associated with higher depressive symptoms. Slowed hippocampal growth has been associated with the development of mental health problems, such as depression, in other human work (see also [84]).

In summary, we suggest that early exposure to adverse, stressful environments *and* positive environments, may result in an initial accelerated maturation of the hippocampus. Larger resulting volumes may promote increased susceptibility to future positive and negative/stressful environmental exposures. Early adverse/stressful exposures, however, result in subsequent reduced hippocampal growth, which leads to increased internalizing symptoms and potentially other poor outcomes. Figure 1 provides an overview of the proposed hippocampal alterations that may occur following adverse and positive environmental exposures across development.

We suggest that there may be similar effects for other brain structures and circuits involved in the detection and processing of salient positive and negative environmental stimuli, including the amygdala and connectivity of the SN. The amygdala is involved in the detection and processing of both positive and negative salient affective stimuli [85] and modulates physiological stress responses [86]. The SN, which involves connections between the amygdala and other midline regions, including the dorsal anterior cingulate cortex, anterior insula, and putamen, is involved in vigilance toward social affective stimuli and, via connections with default mode and cognitive control networks, the generation of appropriate behavioral responses [87].

Existing work partly supports the role of these regions and networks as both mediators and moderators of links between the social environment and mental health in children and adolescents. For example, animal work has shown effects of early adversity on markers of accelerated maturation in the amygdala, similar to those observed in the hippocampus [88]. In humans, adversity has been found to predict larger amygdala volumes in childhood but smaller volumes in adolescence [89], potentially reflecting early acceleration followed by delayed growth. Moreover, increased amygdala reactivity to affective/salient stimuli has been proposed as a differential susceptibility marker [20], and this was supported in the study described above by Liu et al. [58]. Larger amygdala volume has also been found to confer differential susceptibility to depression outcomes in adolescent males [76]. Regarding the SN, Rakesh et al. [27], reviewed above, found that childhood maltreatment was associated with altered development of salience network connectivity. Others have theorized that SN connectivity represents a biomarker for differential susceptibility [90], and this was supported in the study described above by Ding et al. [68]. Further research is needed to test these predictions, including whether they apply to specific types of negative and positive environments, whether chronicity and severity are important factors, when precisely a switch from accelerated maturation to delayed growth occurs, and which specific measures of hippocampal, amygdala, and SN structure, function and connectivity are relevant.

Below, we discuss some of the key limitations in the literature, including areas of theories and models that require further development, in addition to limitations of the current empirical research. Although not comprehensive, we believe they represent important issues that if addressed, will move the field forward considerably.

## Limitations of the current literature, and future directions

### Deficit-focused models and the idea of resilience

With the exception of *differential susceptibility theory* and our proposed *integrated mediation-moderation model*, the prominent focus of the aforementioned models, and much of the empirical literature, has been around risk factors and deficits associated with brain development and mental health. Although such deficit-focused research has provided valuable insights into neurodevelopment and the neurobiological basis of mental illness, it may have oversimplified the complexity of brain and psychological development, with other factors, such as protective factors, often overlooked. Additionally, findings from deficit-focused neurodevelopmental research may contribute to the stigmatization of mental illness, given the emphasis of “problems” associated with mental illness as opposed to acknowledging strengths and potentials for recovery and growth. Shifting focus toward resilience rather than risk could potentially address these limitations.

Recent reviews on the topic of resilience neurobiology have demonstrated that the structure and function of threat, reward, and cognitive control neural networks may be associated with resilience in adults [91–93], that is, associated with positive (or absence of negative) mental health outcomes following adversity. However, our recent systematic review on the subject found that fewer studies have investigated the role of brain development in resilience to poor mental health outcomes in children and adolescents [94].

Further exploration of resilience, particularly in the context of neurobiology, holds promise for informing preventive and therapeutic interventions aimed at promoting mental well-being in youth. It is important to note, however, that while early conceptualizations of resilience suggested it to be an individual trait, this approach has been criticized, including by those suggesting that it may result in power imbalances and discrimination within society [95]. More contemporary conceptualizations emphasize resilience to be a dynamic multisystem process of adaptation post experiences of significant negative experiences; a process that involves brain (and other aspects of biological) development, and other contextual factors at the family, community, and broader levels [96]. Future work, however, is needed to address heterogeneity in the definition of resilience and how best to operationalize and test it [see detailed discussion in [94].

### Adaptation vs. maladaptation

Although the different discussed theories describe both adaptive and maladaptive brain changes, as described above, the current relevant mental health research primarily takes a deficit approach. Any adaptive brain changes identified (in terms of mental health outcomes) tend to be identified “accidentally” (see [94]). Further work is needed, for example, to identify whether brain changes following adversity are adaptive in the short term but maladaptive longer-term, whether they are adaptive versus maladaptive depending on the context (as proposed by *differential susceptibility* and our *integrated mediation-moderation* model), or are adaptive in terms of one outcome but maladaptive in terms of another outcome. Regarding the latter notion, one example of this, which was discussed above, was a finding by Rakesh et al. [27] of increases in SN resting-state functional connectivity to relate to higher depressive symptoms but lower problematic substance use.

Further, regarding adaptive brain changes, there are open questions about whether these brain changes are inherent or driven by supportive environments. Alternatively, they may reflect susceptibility markers, such that later negative and positive experiences propel individuals to negative and positive outcomes, respectively. To comprehensively test differential susceptibility effects, studies are needed that measure positive and negative environments, in addition to positive and negative outcomes. It cannot necessarily be assumed that low levels of a negative environment equate to high levels of a positive environment, for example.

### Type of environment and timing of environmental and brain measures

The vast majority of available studies have investigated the early and mid-adolescent periods, with few studies investigating early and mid-childhood. The early-mid-adolescent period has been suggested as particularly important for the expression of differential susceptibility [97], given that it is associated with increased sensitivity to social stimuli [98] and increased prevalence of psychopathology [5]. Schriber and Guyer [97] suggest that measures of neurobiology may be more likely to reflect susceptibility (sensitivity to both positive and negative environments) than vulnerability (sensitivity only to negative environments) during adolescence relative to other periods (given that adolescence is a period of heightened sensitivity to both negative *and* positive social contexts); future research is needed to test this possibility.

More studies of early-mid childhood, however, are needed to test mediation-based theories, particularly those that focus on the early rearing environment or “early” adversity more generally (including our proposed model). Investigating neurodevelopment immediately or soon after the onset of early adverse experiences will shed light on the initial neurobiological response to adversity. Further, the *change of pace* [26], Rakesh et al.’s model [19], and our proposed *integrated mediation-moderation* model make predictions about patterns of development across childhood and adolescence. Longitudinal studies that capture many years of development are needed to comprehensively test these models.

Relatedly, the majority of studies have not measured the timing of exposure to the social environmental factor of interest. The timing of onset, in addition to the chronicity and severity of exposure, are likely to be important in shaping neurodevelopment or interacting with neurodevelopment to influence mental health [82, 99]. Clarifying timing effects will help to refine models, and more generally determine whether there are specific sensitive periods for different social environmental exposures, and whether these differ for different neural regions and circuits.

Regarding the type of social environment investigated, existing studies have been quite mixed and have investigated varying aspects of the family environment (e.g., childhood maltreatment, family connectedness, parenting behavior), the peer environment (e.g., peer victimization), and broader family, neighborhood, and community-level factors (e.g., family poverty, neighborhood disadvantage, community crime), in addition to general negative life events. Of note, few studies have investigated the peer environment, which may be particularly relevant for *differential susceptibility* effects during adolescence (as per above discussion), or other experiences of social stress, such as minority-related discrimination. There is currently minimal research using mediation frameworks to test differential threat versus deprivation experiences (and how they may interact with unpredictability) as per change of pace theory and the *integrated model of dimensions of environmental experience*. While *differential susceptibility* theory suggests that positive environmental exposures are not merely the inverse of negative environmental exposures [54], not all moderation studies tested positive environmental exposures and hence may have been unable to detect differential susceptibility effects. As such, more research is needed to test whether there is specificity regarding the types of environmental factors and contexts that may influence and interact with neurobiology to predict mental health.

### Integration of human and animal research

Some of the discussed theories and models are, in part, based on evidence from animal research. Animal research, while limited in providing accounts of specific psychiatric disorders, is particularly important for elucidating how different types of environmental exposures causally influence the brain, and how this plays out over time [100]. For example, as outlined above, markers of both accelerated maturation (e.g., precocious arrival of Parvalbumin-positive cells), and growth (e.g., developmental changes in expression of markers of cell proliferation and differentiation) can be investigated over time [82]. Specific drivers of increased neural susceptibility can also be investigated. For example, mouse knockout models have begun to test the function of the SERT polymorphic region (5-HTTLPR) s-allele (which has been linked to differential susceptibility) in inhibitory control over excitatory neurons in the cortex and expression of various GABAergic markers [101].

Animal research will thus be beneficial for testing aspects of the discussed models. Comprehensive animal studies investigating whether and how adverse *and* positive early environments influence neurodevelopmental markers of increased differential susceptibility would be particularly informative. Some relevant work has been done; a recent study, for example, manipulated prenatal stress and rearing-environment quality in prairie voles, and found that prenatal stress was associated with heightened behavioral and physiological reactivity and also with increased susceptibility to both positive and negative rearing experiences [102].

## Conclusion

To conclude, while the role of the social environment in impacting mental health and the development of mental illness in young people has been acknowledged for several decades, knowledge of how neurobiological and neurodevelopmental factors play a role in this lags behind. There have been many theoretical advancements more recently, however, that provide testable hypotheses to move the field forward. A growing research body has started to shed light on the validity of these models. We propose that further research is particularly needed that tests integrated models to provide a comprehensive account of the role of the social environment and neurodevelopment in developmental psychiatry.
